# 3D video tracking analysis reveals that mosquitoes pass more likely through holes in permethrin-treated than in untreated nets

**DOI:** 10.1038/s41598-024-63968-y

**Published:** 2024-06-12

**Authors:** Mathurin Fatou, Pie Müller

**Affiliations:** 1 Swiss Tropical and Public Health Institute, Kreuzstrasse 2, 4123 Allschwil, Switzerland; 2 University of Basel, Petersplatz 1, 4001 Basel, Switzerland

**Keywords:** Malaria, Entomology, Biological techniques

## Abstract

In addition to killing, mosquito nets treated with permethrin have been claimed to repel mosquitoes, reducing their success in passing through a holed net. We have tested this hypothesis by tracking mosquitoes in a modified World Health Organization tunnel test. In the original assay, mosquitoes are released at one end of the tunnel and have to pass through a holed piece of net to reach the bait at the other end. The mosquitoes are left in the tunnel overnight, while mortality and feeding rates are scored the following morning. Since the original test does not reveal how mosquitoes move within the tunnel, we combined the tunnel with a 3D video camera system. We tracked susceptible and permethrin-resistant *Anopheles gambiae s.s.* as they moved in the tunnel and interacted with an untreated or a permethrin-treated net (Olyset Net^®^). Surprisingly, while permethrin increased the mortality and reduced blood-feeding rates, twice as many mosquitoes passed through the holes of the permethrin-treated net. The flight trajectories reveal that upon exposure to the permethrin-treated net, both mosquito colonies showed increased ‘excitation’, thereby augmenting their chance of getting through the holes in the net. The study underlines the complexity of behavioural modes of action of insecticides.

## Introduction

Malaria still causes an estimated 247 million cases globally and results in more than 600,000 deaths per year^1^. To reduce the malaria burden, long-lasting insecticidal nets (LLINs) have been widely distributed and annually avert millions of malaria cases^2^. An ideal LLIN provides personal protection to the user as a physical barrier and through its insecticidal effect as well as indirectly to nonusers, if deployed with high coverage, through an overall reduction in infectious bites.

In addition to insecticide content and bio-efficacy, the long-term efficacy of an LLIN depends on its physical integrity (i.e. the number, size and location of holes in it)^3^. Ideally, an LLIN should provide protection for at least three years, but its integrity may be reduced by physical wear. Physical wear in turn depends on environmental and social factors like climate, type of sleeping space, frequency of use and washing^4^. Long-lasting insecticidal nets are susceptible to hole formation by snag, tear, abrasion, cut, heat, rodents, seam failure, laddering or unravelling^5^ with most holes being usually found along the bottom part of the net^6^.

In addition to killing, the insecticides on LLINs may have other modes of action, such as ’excitation’, ‘irritancy’, ‘repellency’ or a combination (e.g. ‘excito-repellency’^7–11^), albeit there is some confusion over the definition of these terms. Some authors have considered excito-repellency and irritancy being equivalent^12^, others make a distinction between the two terms, whereby irritancy is only upon contact and excito-repellency acts at a distance^11^. With the latter definition, nets treated with excito-repellent insecticides should cause mosquitoes to move away prior to contacting the net^11,13,14^, while irritant insecticides should cause mosquitoes to move away only after contact with the net^15–20^. Multiple studies have reported that permethrin has an excito-repellent effect, resulting in mosquitoes showing avoidance behaviour prior to contact with the permethrin-treated surface^21,22^. In the case of nets treated with permethrin, it has been claimed that the excito-repellent effect would compensate for physical damage as mosquitoes—if neither knocked down nor killed upon contact with the net—would be diverted away^14,23–28^. In contrast, other studies found no evidence for such an excito-repellent effect^9,29–31^. Unfortunately, the terms ‘irritancy’ and ‘excito-repellency’ are problematic and have led to a lot of confusion, since they do not distinguish between the observed outcomes and the underlying mechanisms. They should be replaced by ‘contact disengagement’^12^, unless we have data such as flight trajectories that demonstrate mosquitoes flying indeed away from the stimulus.

The efficacy of a new LLIN formulation is first tested under laboratory conditions, usually in the World Health Organization (WHO) cone bioassay^32^. Nets that meet neither the immediate knockdown (i.e. ≥ 95% knockdown rate) nor the 24 h mortality (i.e. ≥ 80% mortality rate) criterion are then tested in the WHO tunnel test^32^. The WHO tunnel test takes into account behavioural effects such as excito-repellency as the mosquitoes are both less restrained than in the cone test and are exposed to a potential host, which represents a situation that is closer to the real use scenario. In the WHO tunnel test, 100 non-blood-fed female anopheline mosquitoes are released into a 60 cm long tunnel from a cage attached to one end of the tunnel. At the other end is a restrained guinea pig or a rabbit. Two thirds of the way along the tunnel, away from the release cage, a holed net piece is mounted through which the host-seeking mosquitoes have to pass to access the animal host. This brings them into contact with the net and its insecticide. After an exposure of 12–15 h, the mosquitoes’ mortality and blood-feeding rates are recorded. While the WHO tunnel test takes into account mosquito behaviour—in contrast to the WHO cone bioassay—the tunnel test provides only an endpoint measurement and gives no insight into how the mosquitoes actually interact with the insecticide-treated net. Until recently, measuring bed net performance and effects on mosquitoes beyond simple endpoints such as mortality or blood-feeding rates has been technically challenging, but affordable and versatile video camera systems that can record and evaluate the behaviour of free-flying insects—including mosquitoes—are now available^33^.

The aim of the present study is to describe how the malaria vector *An. gambiae s.s.* interacts with a permethrin-treated net (Olyset Net^®^) and test the hypothesis that permethrin prevents host-seeking mosquitoes from passing through a holed net. We combined the WHO tunnel as a flight chamber with a three-dimensional (3D) near-infrared video tracking system to record the flight trajectories of susceptible and insecticide-resistant *An. gambiae s.s.* as they interacted with either a permethrin-treated or an untreated piece of net. Intriguingly, while permethrin increased the mortality in both the susceptible and insecticide-resistant colonies and reduced the blood-feeding rates in the susceptible colony, we found for both colonies that twice as many mosquitoes passed through the holes if the net was treated with permethrin as compared to the untreated net.

## Methods

### Experimental procedure

**Figure 1:**
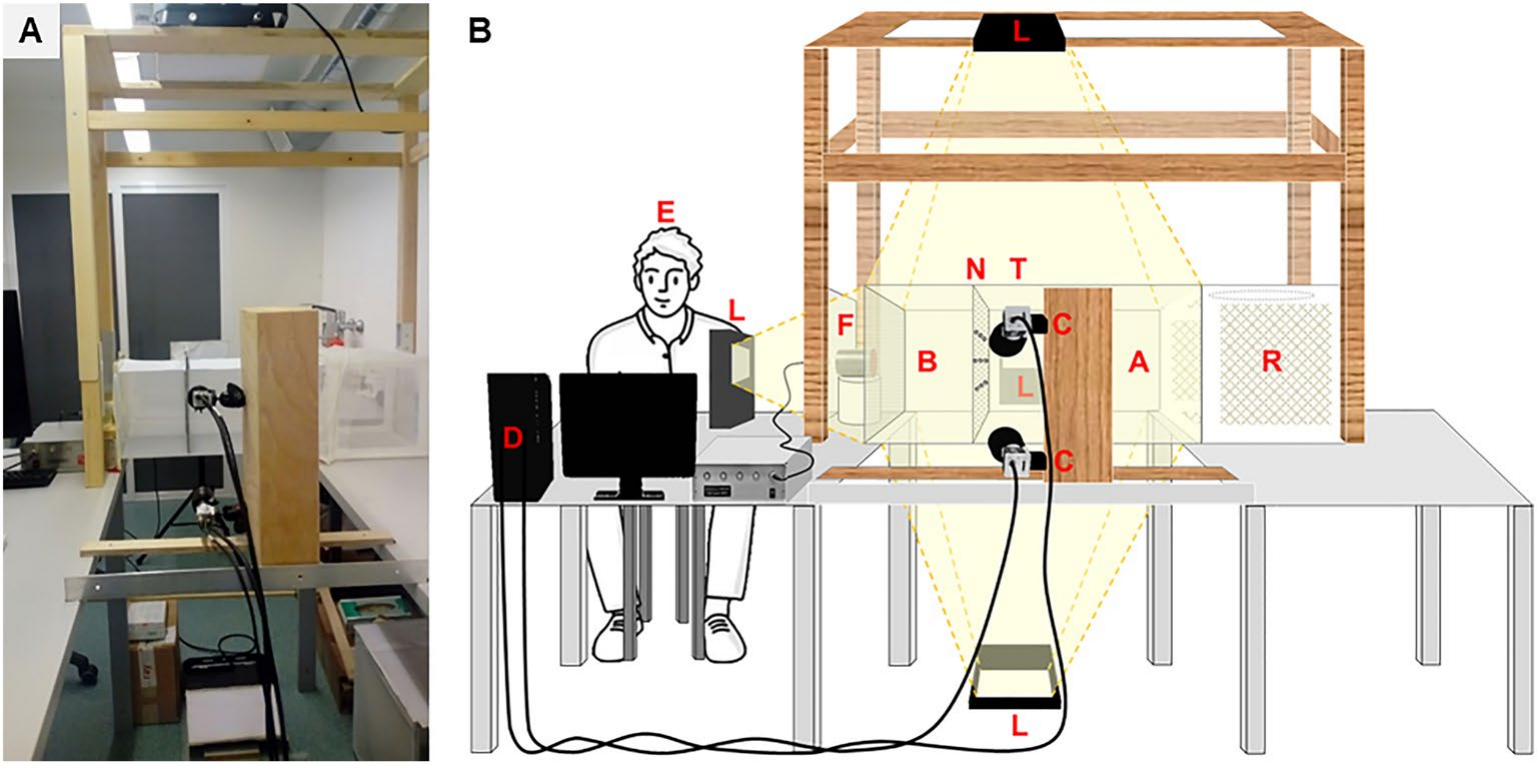
Experimental set-up with the 3D near-infrared video tracking system. (**A**) Original view from behind the cameras' position. (**B**) Sketch of the set-up showing the different elements. The net sample (N) was placed at two thirds of the length of the tunnel away from the release cage (R), dividing the tunnel into a larger (A) and a smaller (B) section. The release cage (R) was attached at the end of the longer section of the tunnel (T), while a blood feeding unit (F) was placed in contact with a mesh closing the end of the shorter section of the tunnel. The two cameras (C) were pointing towards the tracking area, while four NIR lamps (L) illuminated the tunnel. The cameras were connected to a desktop computer and screen (D) placed near the experimental cage, allowing for controlling the system during the experiments. The experimenter (E) sat in front of the computer next to the blood feeding unit.

The experimental procedure was an adaptation of the protocol for the tunnel test described in the WHO guidelines for laboratory and field-testing of LLINs^32^. While the dimensions and the design of the tunnel followed the WHO guidelines, we introduced only one female mosquito at a time into the tunnel, and the guinea pig was replaced by a human, the experimenter (MF), who sat behind the far end of the tunnel, and an artificial feeder from which the mosquitoes could take a blood meal (Fig. 1). The mosquitoes’ flight trajectories were captured with a 3D video tracking system while mosquitoes were exposed to either an untreated or a permethrin-treated net and tracked for 10 min, instead of leaving them in the tunnel for 12–15 h as per WHO guidelines.

### Net samples

We exposed mosquitoes to net pieces with holes from either a permethrin-treated net (Olyset Net^®^, 150 denier polyethylene monofilament, colour white, 20 g/kg permethrin, Sumitomo Chemical Co., Ltd, Tokyo, Japan), with the insecticide being incorporated into the fibre, or to an untreated net (100 denier multi-filament polyester net, colour white).

### Video tracking system

To record the mosquitoes’ flight trajectories, we used the software Trackit3D Fly suite 2.0 (Scitracks GmbH, Bertschikon, Switzerland), installed on a desktop computer (Intel Core i7-8700 CPU @ 3.20 GHz, RAM 16.0 GB, Windows 10). The software was used in combination with two acA2040-90umNIR USB 3 digital cameras (Basler AG, Ahrensburg, Germany), one near-infrared (NIR) light emitting diode (LED) lamp (Raytec pulsestar illuminators PSTR-I96-HV, Raytec Ltd, Ashington, United Kingdom) controlled by a controller (Raytec pulsestar illuminators PSTR-PSU-4CHNL-HV, Ashington, UK), and three custom-made NIR LED lamps (Ingenieurbüro Warnke GmbH, Wietzendorf, Germany).

The video cameras had a resolution of 4 MP and acquired images at a frequency of 50 fps. We equipped the cameras with Fujinon DV3.4×3.8SA-SA1 lenses (Fujifilm Holdings K.K., Tokyo, Japan) and Midopt BP850 NIR band-pass filters (Midwest Optical Systems, Palatine Inc. Illinois, USA) that only pass light at a wavelength above 850 nm. Both lamp types were NIR LED floodlights, emitting light at a wavelength of 850 nm with a power of 70 W. This combination of cameras, lenses, filters and lamps allows for tracking mosquitoes in darkness as mosquitoes are not sensitive to light in the NIR range^34^.

The software of Trackit3D determines the mosquitoes’ positions every 20 ms based on stereo image pairs of dark objects against a bright background by triangulation^35^, while the positional precision is improved through a Kalman filter loop^36^. We calibrated the system with the software’s built-in calibration function, and we set the parameters gain, threshold, exposure time and minimum object area to 0, 9, 4,000 µs and 20 pixels (px), respectively.

In combination with the video tracking software, we used the Open Broadcaster Software version 22.0.2 (Philadelphia Works, Philadelphia, USA) to record videos of the computer screen so that we could later playback the raw images from the two cameras, validating the 3D trajectories where needed, for example, to verify through which hole the mosquitoes passed the net.

### Tunnel experiments

We performed the experiments in a specialised, climate-controlled room at the Swiss Tropical and Public Health Institute (Swiss TPH) in Basel, Switzerland. The design and dimensions of the test tunnel were based on those from the WHO tunnel assay^32^ with slight modifications, allowing for the NIR video tracking system to follow mosquitoes inside the tunnel (Fig. 1).

The external dimensions of the experimental tunnel were 60 cm × 25 cm × 25 cm. The four rectangular panels were made of 5 mm thick acrylic sheets with the top, bottom and back panels being opaque white to produce a homogeneously illuminated background against which the mosquitoes could be tracked as dark objects. One square panel consisted of a sliding acrylic door inserted into a slot, located at one end of the tunnel (to the right in the image shown in Fig. 1). The door panel was attached to an L-shaped wooden handle, allowing to open and close the tunnel manually, in order to control the release of one single mosquito into the tunnel at a time. Attached to the door panel was a 30 cm × 30 cm × 30 cm insect rearing cage (BugDorm-1, MegaView Science Co., Ltd, Taichung, Taiwan) into which we introduced 10 mosquitoes. The release cage had an opening at the top for introducing mosquitoes and another one at the side in contact with the tunnel to allow for releasing mosquitoes individually into the tunnel. The square panel located at the other end of the tunnel (to the left in the image shown in Fig. 1) consisted of a mesh allowing some airflow and mosquitoes to probe through. Here, we placed a Hemotek membrane blood feeding unit (Hemotek Ltd, Blackburn, UK) filled with 36 °C warm, stirred pig blood (Bell Schweiz AG, Basel, Switzerland) covered with parafilm in contact with the mesh on a 12 cm high plastic support outside the tunnel. To track mosquitoes near the blood feeding unit, we placed a piece of white paper between the blood feeder and the PSTR-I96-HV NIR lamp. The paper produces a uniformly bright background against which the darker mosquitoes can be tracked. The net sample, held in a metal frame by magnetic strips, henceforth called ‘net’, was placed at two thirds the length of the tunnel from the release cage, dividing the tunnel into a larger and a smaller compartment (Fig. 1). The tunnel ends rested on two tables separated by 55 cm leaving an open space underneath the tunnel, allowing for illuminating the tunnel from below.

We used 5- to 10-day-old females from the insecticide susceptible *An. gambiae s.s.* Kisumu-1 colony and the *An. gambiae s.s.* Tiassalé colony that is resistant to pyrethroids and other insecticides^37^. The Kisumu-1 colony (MRA-762) was obtained from the Malaria Research and Reference Reagent Center (MR4) in 2011 and has since then been reared as a colony at Swiss TPH. The Tiassalé colony had originally been obtained from the Centre Suisse de Recherches Scientifiques en Côte d’Ivoire in Abidjan and was established at Swiss TPH in 2017. The mosquito colonies were reared in separate rooms to avoid cross-contamination. We maintained the mosquito colonies at 26.7 °C (ranging between 26.4 and 27.1 °C), a relative humidity of 69.9% (ranging between 55.6 and 78.8%) and a day-night-cycle of 12–12 h.

While in the standard WHO tunnel test 100 female mosquitoes are introduced into the tunnel, here, we tracked only one mosquito at a time. Even though the video system can track multiple mosquitoes simultaneously, tracking single mosquitoes allowed us to unambiguously assign all flight trajectories to the same individual as mosquitoes may move outside the cameras’ field of view.

Eight hours before tracking the first mosquito, we removed 10 female mosquitoes from a stock cage in which females and males were allowed to mate and had access to water and a 10% sucrose solution ad libitum. We kept the removed mosquitoes under the same ambient conditions as the stock colony, while they had only access to water without sugar.

We performed experiments with both colonies under subdued red light (peak wavelength of 680 nm) at an intensity of 50 Lux to simulate the conditions according to the mosquito species’ nocturnal biting pattern while providing some light necessary for mosquito behaviour to occur^38^, and to allow the experimenter to operate the experimental set-up. The room with the experimental set-up had an average temperature of 25.9 °C (ranging between 24.9 and 27.5 °C), an average relative humidity of 68.6% (ranging between 53.4 and 83.1%) and an average CO_2_ concentration of 564 ppm (ranging between 478 and 636 ppm). Ambient temperature, humidity and CO_2_ concentration were constantly monitored with Tinytag data loggers (Gemini Data Loggers Ltd, Chichester, UK).

Prior to the experiment, we cut the net into pieces of 25 cm × 25 cm. We holed each net piece with nine 1 cm-wide holes arranged in a rectangle; with one hole in the centre and the other eight holes equidistant from each other and 5 cm from the border following the WHO guidelines^32^. We then placed the net pieces into the frame providing a netting surface area of 20 cm × 20 cm to the mosquitoes.

At 16:00 h, 30 min before the start of tracking the first mosquito, we introduced 10 starved mosquitoes into the release cage for acclimation. The Hemotek feeding unit was filled with 1 ml blood, covered with parafilm, connected to a Hemotek PS6 Power Unit to keep the blood at 36 °C, and placed in contact with the mesh at the end of the tunnel. We then inserted the holed net sample into the tunnel, while we had previously randomised the order of placement of net type using the function ‘sample’ in R version 4.1.2^39^.

In addition of releasing one single mosquito at a time from the release cage, we also adapted the standard WHO protocol in that we left the released mosquito for only 10 min in the tunnel instead of overnight for 12–15 h. This allowed for running 10 replicates per day, since preliminary experiments showed the time to be sufficient for a mosquito to reach the other end of the tunnel and to blood-feed under normal circumstances.

At 16:30 h, we started the video tracking for the first of 10 mosquitoes. The experimenter pulled the handle to release a single mosquito into the tunnel and blew twice into the cage to provide CO_2_ and odour stimuli, triggering the mosquito to initiate host seeking^40^. Once the mosquito entered the tunnel, the experimenter closed the release cage again to keep the remaining mosquitoes in the cage. At the end of the 10-min exposure, he stopped the video tracking and removed the mosquito from the tunnel. We repeated the procedure until five mosquitoes were exposed to the same net piece. We then removed the net from the tunnel, replaced it with the other net and repeated the same procedure with the remaining five mosquitoes. Thus, from the batch of 10 mosquitoes, five were exposed to the permethrin-treated and five to the untreated net. We repeated the experiments until we had exposed 50 mosquitoes from each colony and to each net type. Before changing nets, and regardless which net was used first, we washed the tunnel with neutral soap to remove any insecticide residues from the tunnel.

### Data analysis

The readouts of the tracking system were Cartesian *x*-, *y*- and *z*-coordinates with a time stamp at a temporal resolution of 50 Hz. We stored the data as CSV files on the hard drive of the desktop computer while the positional data were already filtered for outliers and missing observations. We imported the data from the CSV files into R version 4.1.3^39^ for data analysis. For creating the plots, we used either the ‘rgl’^41^ or the ‘ggplot’^42^ package.

As the data points correspond to a discrete time series with one measurement every 20 ms, we applied the finite helix fit (FHF) approach of Crenshaw et al.^43^ that has previously been applied to analyse flight trajectories of tachinid flies^44^. The FHF reconstructs a 3D trajectory by decomposing the trajectory into its translational velocity ***V***, with speed *s*, curvature ***κ*** and torsion ***τ*** as functions of time.

For the analysis of the spatial distribution of mosquito activity at the net interface, missing positions of mosquitoes were first filled with the last tracked position using the ‘na.locf’ function from the R ‘zoo’ package^45^. We then virtually divided the net surface and the frame area around the net into a grid with 5 mm × 5 mm raster cells (i.e. ‘pixels’). From the pixels, we created heat maps for visualisation and data analysis purposes by counting the time spent by each mosquito within each pixel. We considered mosquitoes being in contact with the net if they were within 3 mm of the virtual position of the net. Using the R package ‘spatstat’^46^, we also measured the clustering of the mosquitoes’ net contacts by transforming our data frames into point patterns and performing a nearest neighbour measure Clark-Evans test^47^. The Clark and Evans aggregation index *R* is a crude measure of clustering or ordering of a point pattern. It is the ratio of the observed mean nearest neighbour distance in the pattern to that expected for a Poisson point process of the same intensity. A value *R* > 1 suggests ordering, while *R* < 1 suggests clustering.

We calculated mortality, blood feeding and net passage rates as the proportions of dead, blood-fed and mosquitoes that had passed through the net, respectively, divided by the number of mosquitoes that approached the net. For net passage, we only counted the first passage through the net, since occasionally mosquitoes went back again through the net. To assess differences in mortality, blood feeding, net passage rates as well as the mosquitoes’ preference to land at the upper or lower, and left or right, half of the net, we estimated the odds ratios and their 95% confidence intervals (95% CI) between the permethrin-treated and the untreated net for each mosquito colony. The estimates were based on generalised linear models (GLMs) with a binomial error distribution and a logit link function using the R function ‘glm’ in the ‘stats’ package^39^.

To assess the differences in flight velocity, curvature and torsion as well as the time spent in contact with the net, the average height at the net, and the average surface coverage, we fitted linear models using the function ‘lm’ from the R ‘stats’ package. For the average time spent at the net, we log-transformed the time, since its distribution was skewed towards the lower end. We used the ‘offset’ function in the model to compare the average surface coverage of the net as a function of the average time spent at the net. Similarly, we log-transformed curvature and torsion.

To assess overall changes in the number of mosquitoes that passed through each hole between the permethrin-treated and untreated nets, we used the Fisher’s exact test. For significance testing of the terms in the GLMs, we ran ANOVAs comparing the reduced models with the full models.

We set the level of statistical significance at *α* = 0.05.

## Results

### Mortality, blood-feeding and net passage rates

In the original WHO tunnel test, the efficacy of LLINs is measured by the proportion of dead and blood-fed females following an exposure time of 12 to 15 h. Although the mosquitoes were not left over night, we also calculated those endpoints. As the mosquitoes were tracked, we could also count the number of mosquitoes that passed through the holes of the nets.

Upon opening the door of the release cage, most of the individually released mosquitoes immediately entered the tunnel and flew towards the net. Out of 50 mosquitoes per colony and net type, 47 susceptible mosquitoes entered the tunnel and flew towards the untreated net, 43 susceptible mosquitoes approached the permethrin-treated net, 43 resistant mosquitoes flew towards the untreated net, and 45 resistant mosquitoes flew to the permethrin-treated net.

**Figure 2:**
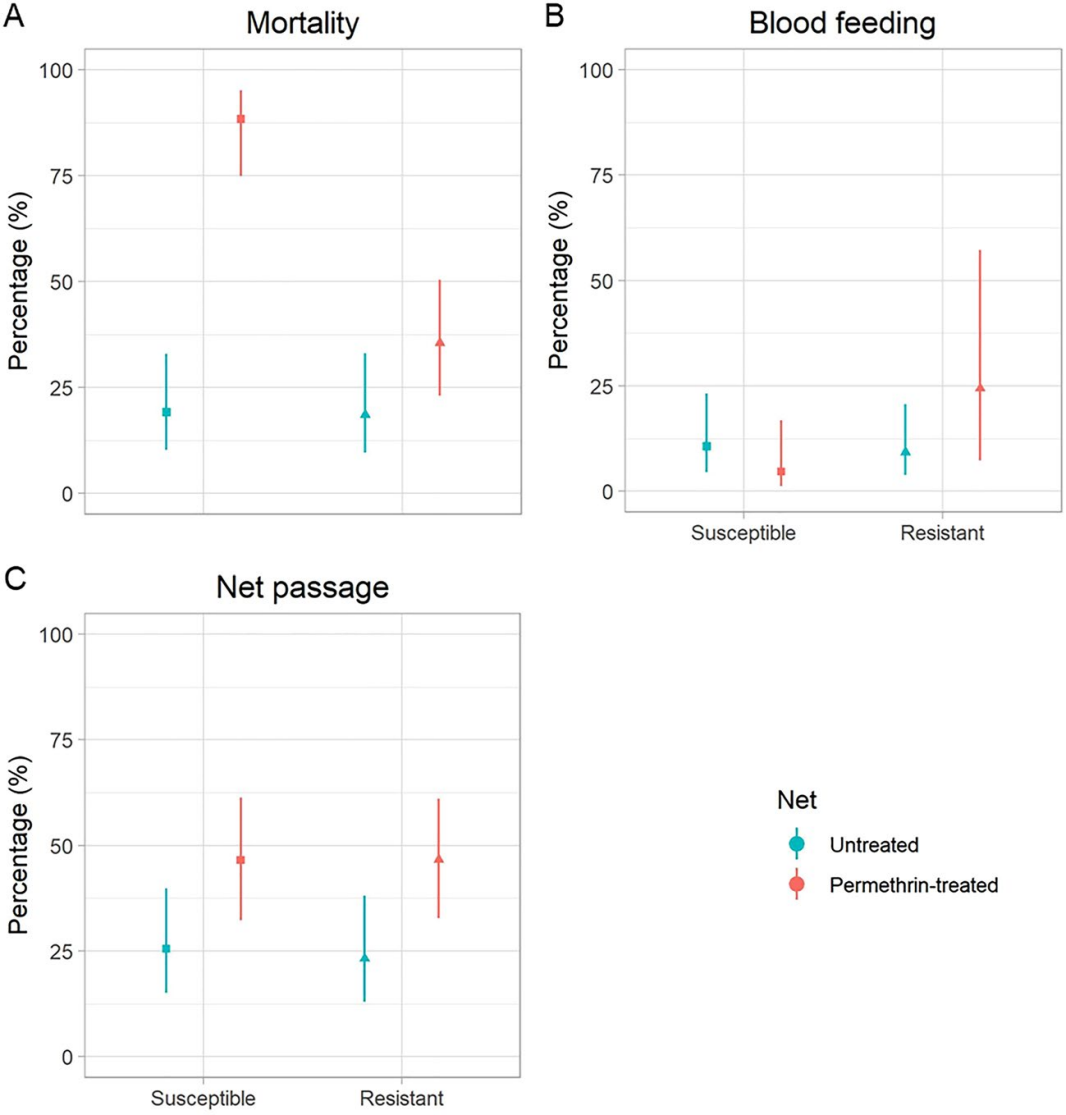
Change in mortality, blood feeding and net passage rates in insecticide susceptible and resistant *Anopheles gambiae s.s.* exposed to a permethrin-treated versus an untreated net. (**A**) Delayed mortality measured 24 h after removing the mosquitoes from the tunnel. (**B**) Blood feeding rates at the end of the experiments. (**C**) Rates of mosquitoes passing through the holes of the nets. The dots show the average percentage, while the vertical lines indicate the 95% confidence intervals that were estimated with a generalised linear model with a logit-link function and a binomial error distribution.

Mortality in the susceptible colony was significantly higher for the mosquitoes exposed to the permethrin-treated net than those exposed to the untreated net with an odds ratio (OR) of 32.1 (95% CI 9.8–104.6), while this difference was statistically not significant in the resistant colony (OR 2.4; 95% CI 0.9–6.4; Fig. 2a). The differences in the blood feeding rates were statistically not significantly different neither for the susceptible (OR 0.4, 95% CI 0.1–2.2) nor the resistant colony (OR 3.2, 95% CI 0.9–10.8) (Fig. 2b). Contrary to expectations, more mosquitoes passed through the permethrin-treated net—regardless of the colony—with an OR of 2.5 (95% CI 1.0–6.2) and 2.9 (95% CI 1.2–7.2) for the susceptible and insecticide-resistant colony, respectively (Fig. 2c).

### Mosquito trajectories

**Figure 3:**
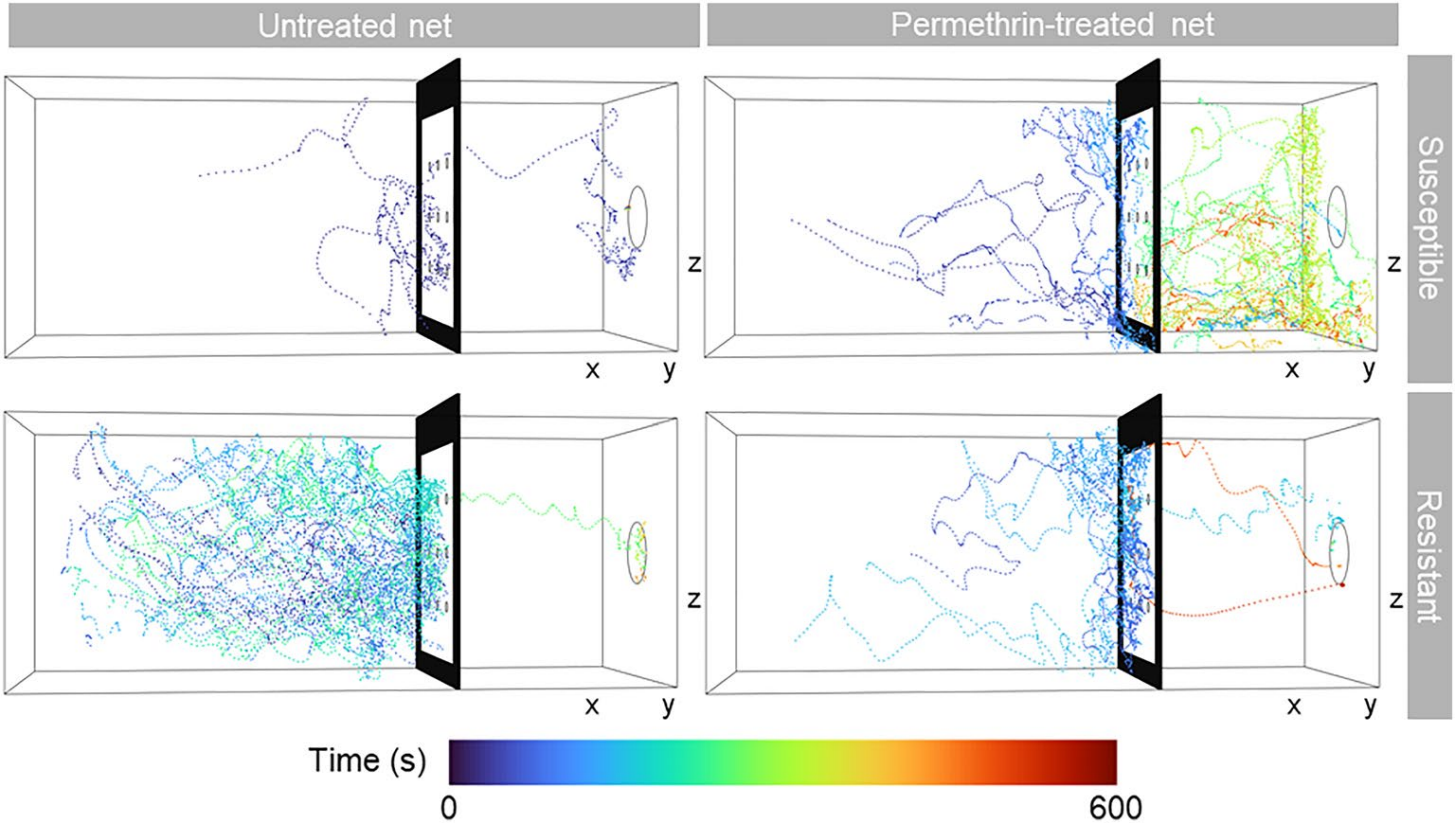
Examples of 3D flight trajectories of insecticide susceptible and resistant *Anopheles gambiae s.s.* exposed to a permethrin-treated or an untreated net. Each panel shows the flight trajectory of a single mosquito recorded during 10 min (i.e. 600 s). The frame holding the net is represented by the black rectangle. The circle on the right hand side of the plots corresponds to the position and size of the blood-feeding unit. All four examples are from mosquitoes that landed on and fed through the membrane of the blood-feeding unit.

We recorded flight trajectories for 10 min from each of 178 mosquitoes. With a sampling frequency of 50 Hz this corresponds to 30,000 *xyz* positional data points per mosquito and more than 5 M data points in total. However, the tracking system could not always track the position of the mosquito, primarily when mosquitoes left the field of view by flying or resting close to the tunnel entrance, or when they were in front of the blood feeding unit, since the contrast of the mosquito against the unit was poor. However, within the area of interest between the net and 20 cm in front of the net, 78% (minimum: 63%; maximum: 87%) of the mosquitoes’ positions were successfully tracked, including both flying and non-moving mosquitoes (Fig. 3; Supplementary video S1).

Figure 3 shows examples of flight trajectories for each of the four combinations of net type and mosquito colony. Upon entering the tunnel, the mosquitoes flew towards the net and, rather than flying straight through a hole, they typically had multiple contacts with the net. The contacts were characterised as either isolated events or as bouncing^48^ along the net. Once the mosquitoes got through a hole of an untreated net, they immediately approached the blood feeding unit and remained there until the end of the 10 min observation window. In contrast, when the net was treated with permethrin, mosquitoes from both colonies left the feeding unit earlier.

We estimated the total time mosquitoes were moving as well as the time it took them to pass through a hole in the net. During most of the 10 min observation period the mosquitoes were not moving. On average, mosquitoes from the insecticide susceptible colony moved for 201.4 s (95% CI 170.7–232.1 s) when exposed to an untreated net and about half as long when exposed to a permethrin-treated net (mean: 132.4 s; 95% CI 100.0–164.8 s; *t* = 3.874, *p* < 0.001). In contrast, the reduction in movement was not statistically significantly different for the insecticide resistant colony (*t* = 1.337, *p* = 0.185).

In contrast to the overall movement, the time required to pass through a hole was statistically not significantly different across nets and mosquito colonies. The mosquitoes from the insecticide susceptible colony passed through the holes of the untreated and permethrin-treated net within 202.0 s (95% CI 98.7–305.3 s) and 187.8 s (95% CI 117.4–258.1 s), respectively, while the mosquitoes from the resistant colony passed through the nets in 264.2 s (95% CI 159.7–368.7 s) and 213.6 s (95% CI 141.7–285.4 s), respectively.

### Speed, curvature and torsion

**Figure 4:**
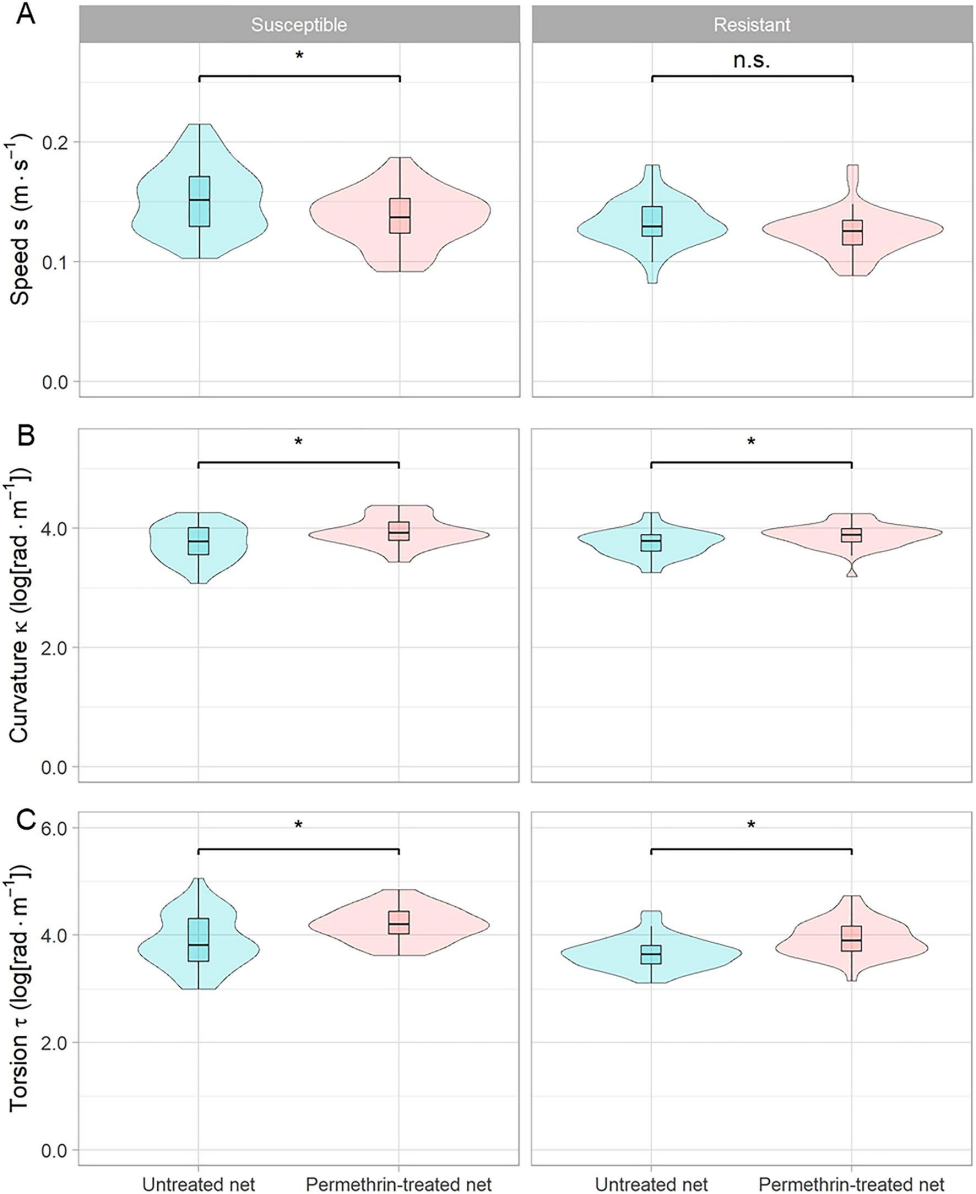
Estimates of speed, curvature and torsion of mosquito flight trajectories between insecticide susceptible phenotypes and net types. Each parameter was estimated by the finite-helix-fit approach. Torsion is presented in absolute values as otherwise positive and negative values would average to zero. The *p*-values were estimated with separate linear models with speed, curvature and torsion as the outcome variables while curvature and torsion were log-transformed. Net type and mosquito colony were included as fixed terms and the error distributions were assumed to follow a normal distribution. *p < 0.05. n.s.: not significant.

Mean speed, curvature and torsion of the flight trajectories may inform if and how mosquito behaviour is influenced by the net treatment and mosquito colony as the flight trajectories are defined by these three parameters. We investigated these parameters over the whole duration of each test (i.e. 600 s) to see if there was an effect of net treatment (Fig. 4).

When exposed to an untreated net, the susceptible mosquitoes flew faster than the resistant ones (*F*_1, 88_ = 15.5, *p* < 0.001) with average speeds of 0.15 m s^−1^ (95% CI 0.14–0.16 m s^−1^) and 0.13 m s^−1^ (95% CI 0.13–0.14 m s^−1^), respectively (Fig. 4a). Similarly, when exposed to a permethrin-treated net, the susceptible mosquitoes flew faster than the resistant ones (*F*_1, 86_ = 8.1, *p* = 0.006) with average flight speeds of 0.14 m s^−1^ (95% CI 0.13–0.14 m s^−1^) and 0.12 m s^−1^ (95% CI 0.12–0.13 m s^−1^), respectively. Nevertheless, the average flight speed in the susceptible mosquitoes was reduced when exposed to the permethrin-treated net as compared to the untreated net (*F*_1, 88_ = 8.089*, p* = 0.006). Though statistically not significant, there was also a tendency in the resistant mosquitoes to fly more slowly when exposed to the permethrin-treated net (*F*_1, 86_ = 3.825, *p* = 0.054).

The average curvature in the insecticide susceptible colony was 3.8 rad s^−1^ (95% CI 3.7–3.8 rad s^−1^) when exposed to the untreated net (Fig. 4b). For susceptible mosquitoes exposed to the permethrin-treated net, curvature increased to 3.9 rad s^−1^ (95% CI 3.9–4.0 rad s^−1^; *F*_1, 88_ = 11.19, *p* < 0.001). A similar picture was observed in the insecticide-resistant colony for which the curvature increased from 3.7 rad s^−1^ (95% CI 3.7–3.8 rad s^−1^) to 3.9 rad s^−1^ (95% CI 3.8–3.9 rad s^−1^; *F*_1, 86_ = 8.408, *p* = 0.005) when exposed to the untreated and the permethrin-treated net, respectively. In addition, there was no statistically significant difference between the colonies when exposed to either an untreated (*F*_1, 88_ = 0.005, *p* = 0.942) or a permethrin-treated net (*F*_1, 86_ = 2.278, *p* = 0.135).

In contrast to curvature, there was a statistically significant difference in torsion between colonies for both net types (*F*_1, 88_ = 6.528, *p* = 0.012 and *F*_1, 86_ = 15.67, *p* < 0.001), while the average torsion of the flight trajectory in both colonies was slightly greater for a permethrin-treated versus an untreated net (Fig. 4c). In the susceptible colony, average torsion decreased from 4.2 rad s^−1^ (95% CI 4.1–4.3 rad s^−1^) to 3.9 rad s^−1^ (95% CI 3.7–4.0 rad s^−1^; *F*_1, 88_ = 13.08, *p* < 0.001) for the permethrin-treated and untreated net condition, respectively. For the resistant colony, torsion decreased from 3.9 rad s^−1^ (95% CI 3.8–4.0 rad s^−1^) to 3.7 rad s^−1^ (95% CI 3.6–3.7 rad s^−1^), respectively (*F*_1, 86_ = 17.14, *p* < 0.001).

### Activity at the net surface

Overall, mosquitoes spent more time at the permethrin-treated than at the untreated net (*F*_2, 174_ = 7.447, *p* < 0.001) with a much more pronounced difference seen in the insecticide-resistant colony (Table 1). However, irrespective of the net type and mosquito colony, the mosquitoes spent about the same proportion of time moving and being stationary on the net.

**Table 1:** Mosquito activity at the net surface.

Colony	Net	*n*	Total time (s)	Proportion moving
Susceptible	Untreated	47	15.8 (9.4–26.5)	0.45 (0.36–0.57)
	Permethrin-treated	43	21.5 (12.5–36.8)	0.45 (0.35–0.57)
Resistant	Untreated	43	2.4 (1.3–4.5)	0.61 (0.43–0.84)
	Permethrin-treated	45	11.7 (6.3–22.0)	0.42 (0.31–0.59)

Total time: duration a mosquito spent at the net surface. Proportion moving: proportion of the total time a mosquito was moving at the net. The averages and the 95% confidence intervals (values in brackets) of the total time spent at the net and the proportions of time moving were estimated using linear models with time being log-transformed and a normal error distribution.

The net area covered by the mosquitoes, defined as the number of 5 mm × 5 mm pixels occupied across the net at least once, was 169 px (95% CI 127–211 px) in the susceptible colony on the untreated net and 148 px (95% CI 114–183 px) on the treated net (*F*_1, 88_ = 0.232, *p* = 0.632). In contrast, the resistant mosquitoes showed a tendency to occupy a larger area when exposed to the permethrin-treated net, covering on average 118 px (95% CI 91–145 px) versus 80 px (95% CI 53–108 px), respectively (*F*_1, 86_ = 3.826, *p* = 0.054).

**Figure 5:**
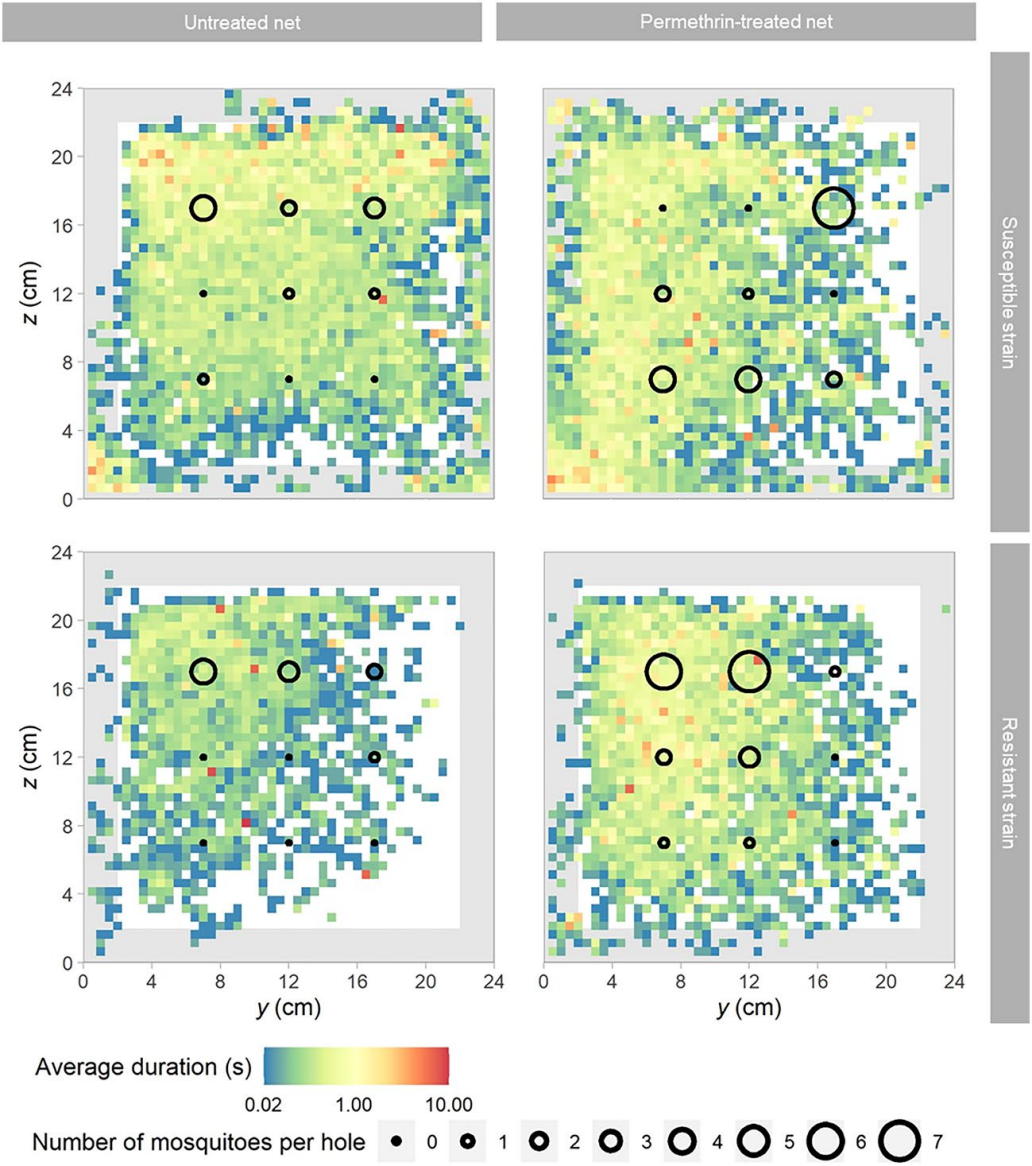
Activity pattern on the net between mosquito colonies and net types. The heat map illustrates the average duration a mosquito spent at a given location on the side facing the entrance of the tunnel. Each pixel represents an area of 5 mm × 5 mm on the net and its colour corresponds to the average duration a mosquito spent within that area, irrespective if the mosquito was stationary or moving along the net. Note that the average duration is presented on the natural log scale. White pixels indicate absence of mosquitoes, and the grey shaded area represents the holding frame of the net piece. Black circles are centred at the positions of the holes in the net with their size being proportional to the number of mosquitoes that passed through that hole.

While there was no apparent difference when they were exposed to the permethrin treated net, mosquitoes from the susceptible colony went through the holes in the upper row of the untreated net more frequently than through the holes in the lower two rows (Fig. 5; Fisher’s exact test, *p* = 0.025). This effect is reflected by a reduction in the average height of the susceptible mosquitoes when active at the net with the frame excluded (*F*_1, 88_ = 12.21, *p* < 0.001). On the untreated net, the height was 12.0 cm (95% CI 11.9–12.0 cm), while on the permethrin-treated net the mosquitoes’ average height was 10.1 cm (95% CI 9.9–10.1 cm). In contrast, the average height of the resistant mosquitoes was similar (*F*_1, 86_ = 1.047, *p* = 0.309) on both nets (i.e. untreated net = 12.7 cm, 95% CI 12.6–12.8 cm; permethrin-treated net = 10.0 cm, 95% CI 9.8–10.0 cm). Of the 63 mosquitoes that flew through a hole in the net, five flew back again through a hole.

## Discussion

While the endpoints of the standard WHO tunnel assay, mortality and blood-feeding rates, are instructive for the efficacy of an insecticide-treated net, these endpoints reveal little about the behavioural mode of action of the active ingredient, and how mosquitoes interact with the net. Therefore, we modified the WHO tunnel assay and combined it with a 3D video tracking system to follow the flight paths of susceptible and permethrin-resistant mosquitoes as they interact with either an untreated or a permethrin-treated, holed net. We then tested whether a permethrin-treated net repels mosquitoes. As expected, we found that exposure to the permethrin-treated net markedly increased the mortality in the susceptible colony and reduced the time spent blood-feeding in comparison to mortality and blood-feeding in the presence of an untreated net. Surprisingly, individuals from both mosquito colonies were more likely to pass through the holes in the net when the net was treated with permethrin. Our results do not support the hypothesis that pyrethroids protect a holed net from mosquitoes passing through and provide no evidence for permethrin being repellent.

While, in the presence of the permethrin-treated net, the susceptible mosquitoes spent more time moving around at the net surface and lowered their average flight altitude—instead of flying towards the top of the net—mosquitoes from the resistant colony tended to cover a larger area while being more active on the net. In addition, mosquitoes from both colonies reduced their flight speed, while their trajectories were more tortuous when exposed to the permethrin-treated net. Together, this may explain why mosquitoes from both colonies were more likely to pass through one of the holes in the permethrin-treated than in the untreated net. It has previously been shown that the behaviour of *An. gambiae s.s.* and the position of a hole in the net affects a mosquito’s chance in getting through^49^. Similarly, the intensified mobility observed here could have increased the chance of mosquitoes encountering the holes and, subsequently, passing through them.

Previous studies have shown contradictory results for the behavioural effects of permethrin. While some studies suggest a repellent effect on mosquitoes at close range^21,22^, others did not find such an effect^9,29–31,50^. Lindsay et al.^30^ found that the repellent effect of permethrin-treated nets was linked to the emulsifier and not to the insecticide itself, which might have led to the wrong notion that permethrin is repellent. Here, the permethrin was already incorporated into the fibre of the net, a technique commonly used for LLINs. Discrepancies between studies may also arise from the problem with the interpretation of behavioural endpoints from bioassays. These bioassays do not distinguish between the underlying behavioural mechanisms leading to those endpoints. A recent room-scale infrared video tracking study in *An. gambiae s.l.,* testing the same net as here, also failed to find a repellent effect^51^. Similarly, when using video tracking analysis of *An. gambiae s.s.* in response to a deltamethrin-treated net in a wind tunnel, Spitzen et al.^52^ did neither see a repellent effect. Behavioural outcomes may also be context-dependent. Hauser et al.^53^ found that contact with a permethrin-treated net did not prevent *An. gambiae s.s.* from blood feeding on a human host if mosquitoes could reach the skin through the net. In contrast, holding an arm behind the permethrin-treated net beyond the mosquitoes’ probing range elicited an excitatory effect, or contact disengagement effect according to the nomenclature of Miller et al.^12^, in both susceptible and resistant *An. gambiae s.l.*^54^. We found a similar effect in our experiment, since mosquitoes from both the insecticide susceptible and resistant colonies showed increased displacement after contact with the permethrin-treated net while being exposed to host cues, albeit the effect was stronger in the susceptible colony.

A limitation of the present study is that the experimental set-up is a simplified model of the real use scenario with an entire net. Studies with entire nets have shown that *An. gambiae s.l.* mosquitoes may interact with the net for more than 10 min, while we stopped the experiment at 10 min. However, mosquitoes were found to be mostly active above the human host^48,55,56^, where they encounter horizontally-oriented holes in the net roof. In contrast, in the present study, the mosquitoes were confined to approach the blood-feeding unit from the side and passing through vertically-oriented holes. The position and the number of holes in the netting sample has most likely influenced the outcome. The mosquitoes had alternative holes to pass through the net when they flew lower (i.e. when they were affected by the permethrin). Still, while our set-up only partially mimics a real world scenario, mosquito nets tend to be often damaged at the bottom^6,57^; hence, if mosquitoes do drop upon contact with a permethrin-treated net, our observations are still relevant.

Our findings challenge the idea of permethrin acting as a repellent by having measured the actual behaviour of mosquitoes rather than drawing conclusions from bioassay endpoints alone (i.e. mortality and blood-feeding rates). Understanding the behavioural effects of vector control tools by measuring the actual behaviour of mosquitoes rather than relying solely on simple endpoints is, therefore, important and will help improving the delivery and evaluation of new active ingredients, particularly those that have additional or alternative modes of actions to killing. Moreover, understanding the behaviour may also explain why a product performs differently in various contexts as seen, for example, in data generated from different experimental hut studies with the same product^58^. Therefore, it would be desirable to perform more studies around LLINs inside and outside semi-field set-ups, similar to the studies of Parker et al.^56^ and Spitzen et al.^50^ using 3D video tracking, to better understand mosquito behaviour in a setting that is closer to the real use scenario. This would also allow comparisons of how well the WHO tunnel test set-up reflects the ‘real world’; hence, making evidence-based recommendations to improve LLIN formulations and efficacy testing.

In conclusion, in our study, permethrin has little or no repellent effect and does not reduce the number of mosquitoes passing through a holed net within a short time frame. The 3D flight trajectories suggest that upon exposure to a permethrin-treated net, both insecticide-susceptible and resistant *An. gambiae s.s.* show increased contact disengagement—albeit at different levels—thereby augmenting their chance of encountering and passing through the holes in the net. The study underlines the complexity of the behavioural effects of insecticides and the importance of measuring behaviour beyond simple endpoints with methods such as 3D video tracking.

### Supplementary Information

Supplementary Information.

Supplementary Information.

## Data Availability

The datasets generated and analysed during the current study are available from the corresponding author on reasonable request.
