# Supplementary material for: 3D video tracking analysis reveals that mosquitoes pass more likely through holes in permethrin-treated than in untreated nets
Source: Sci Rep. 2024 Jun 12;14:13598. doi: 10.1038/s41598-024-63968-y (PMC11169678; doi:10.1038/s41598-024-63968-y)
Supplement: Supplementary file 1 — Supplementary Information. [file 41598_2024_63968_MOESM1_ESM.docx]

**Supplementary Video S1: Extracts of tunnel assays with each colony and net type.** This video shows examples of susceptible and permethrin-resistant mosquitoes participating in tunnel experiments and interacting with an untreated or a permethrin-treated net. Out of the 10 min experiments, representative 30 s clips are shown and played at actual speed.
